# Special Issue Second Call: Plant‐environment interactions in Africa—Solutions to the challenges of environmental change

**DOI:** 10.1002/pei3.10085

**Published:** 2022-06-29

**Authors:** Wayne Dawson, Stacy Singer, Abdelbagi Ismail

**Affiliations:** ^1^ Department of Biosciences Durham University Durham UK; ^2^ Agriculture and Agri‐Food Canada Lethbridge Research and Development Centre Lethbridge Alberta Canada; ^3^ International Rice Research Institute‐Africa Nairobi Kenya

We have been delighted to receive and read manuscripts that authors have submitted for our upcoming Special Issue focused on plant‐environment interactions in Africa. However, we are still keen to receive and consider additional manuscripts from eligible authors. Therefore, **the deadline for submitting manuscripts for this Special Issue has been extended to the 31st of August 2022.**

There are two main motivations behind creating this Africa‐focused Special Issue in P‐EI. First, we recognise that environmental change poses acute and wide‐ranging challenges to agriculture, forestry, ecological systems and plant biodiversity in African countries. Second, we also recognise that researchers in the Global South, and especially in African countries, are under‐represented in the international plant and environmental sciences literature. For these reasons, we would like this Special Issue to promote the science of African researchers in these fields, especially work with a focus on environmental change. **Environmental change is meant in its broadest sense, and can include (but is not restricted to): climate change, habitat degradation and destruction, land‐use change, pollution, invasive species, overexploitation, urbanization, desertification, and salinization.**

Articles can be from across the scope of the plant and environmental sciences that we normally consider for publication, including plant ecology and evolution, biodiversity, plant physiology and crop science, plant responses to biotic and abiotic stresses, and cell and molecular biology. We will also consider literature reviews, and shorter, more exploratory manuscripts under our “Interaction Insights” category. The research and the lead author or corresponding author should be based in Africa. All accepted publications should meet our four key criteria (please see our author guidelines), but the most important criterion is that manuscripts should be focused on interactions between plants and their environment (‘environment’ is also meant in its broadest sense and can include abiotic or biotic environmental conditions, or both).

All manuscripts submitted for consideration in this Special Issue will be subject to initial screening and if deemed suitable, will be subject to peer review. **All articles in this Special Issue will be published without cost to the contributing authors.**

Please email or the Editor‐In‐Chief (wayne.dawson@durham.ac.uk).

We look forward to receiving more of your submissions!

